# Demographic, Virological Characteristics and Prognosis of Asymptomatic COVID-19 Patients in South China

**DOI:** 10.3389/fmed.2022.830942

**Published:** 2022-01-28

**Authors:** Hui Xu, Cheng-yuan Xie, Pei-hong Li, Zhong-liang Ji, Jiu-feng Sun, Bei Hu, Xin Li, Ming Fang

**Affiliations:** ^1^Guangdong Provincial People's Hospital, Guangdong Academy of Medical Sciences, Guangzhou, China; ^2^The Second School of Clinical Medicine, Southern Medical University, Guangzhou, China; ^3^Shantou University Medical College, Shantou, China; ^4^Department of Emergency, Shenzhen University General Hospital, Shenzhen, China; ^5^Guangdong Provincial Institute of Public Health, Guangdong Provincial Center for Disease Control and Prevention, Guangzhou, China

**Keywords:** COVID-19, asymptomatic patients, variants, prognosis, epidemiology

## Abstract

**Background:**

Asymptomatic transmission is a major concern for SARS-CoV-2 community spread; however, little information is available on demographic, virological characteristics and prognosis of asymptomatic cases.

**Methods:**

All COVID-19 patients hospitalized in Guangdong Province from September 1, 2020 to February 28, 2021, were included and were divided into asymptomatic and symptomaticgroup. The source country of all patients, clinical laboratory test results, the genotype of virus and the time of SARS-CoV-2 RNA turning negative or hospitalization were confirmed.

**Results:**

Total 233 patients from 57 different countries or regions were included, with 83 (35.6%) asymptomatic and 150 (64.4%) symptomatic patients. Asymptomatic cases were younger (*P* = 0.019), lower rate in comorbidities (*P* = 0.021) such as hypertension (*P* = 0.083) and chronic liver disease (*P* = 0.045), lower PCT (*P* = 0.021), DDI (*P* < 0.001) and ALT (*P* = 0.029), but higher WBC count (*P* = 0.002) and lymphocyte (*P* = 0.011) than symptomatic patients. As for SARS-CoV-2 subtypes, patients infected with B.1.1 (53.8%), B.1.351 (81.8%) and B.1.524 (60%) are mainly asymptomatic, while infected with B, B.1, B.1.1.63, B.1.1.7, B.1.36, B.1.36.1, B.1.36.16, B.1.5 and B.6 were inclined to be symptomatic. Patients infected with variant B.1.351 and B.1.524 spent longer time in SARS-CoV-2 RNA turn negative (26 days, *P* = 0.085; 41 days, *P* = 0.007) and hospitalization (28 days, *P* = 0.085; 43 days, *P* = 0.004).

**Conclusions:**

The asymptomatic cases are prone to develop in patients with younger age, less comorbidities andinfected with B.1.1 and B.1.524 variants. More attention should be paid for lineage B.1.524 because it can significantly prolong the SARS-CoV-2 RNA negative conversion time and hospitalization in infected cases.

## Introduction

The Coronavirus disease 2019 (COVID-19) is still a worldwide pandemic. Recently, there have been many clusters of cases in China, such as Guangzhou, Nanjing, Heilongjiang, Shaanxi, Chongqing and Jiangsu, among which asymptomatic cases account for a large proportion. “Asymptomatic” is defined as an individual with laboratory-confirmed SARS-CoV-2 infection but without symptoms throughout their entire course of infection, or after 14 days follow-up (1). Currently asymptomatic transmission is a major concern for SARS-CoV-2 community spread (1). Some studies indicate that the asymptomatic persons can indeed transmit SARS-CoV-2 to others which cause the rapid spread of the virus (2). Moreover, the asymptomatic persons are likely to have far more interaction with others than those who have symptoms (2). There are a large number of asymptomatic infections in the world. Through an analysis of more than 350 clinical studies, 42.8% of cases exhibited no symptoms, a group comprising both asymptomatic and presymptomatic infections, and truly asymptomatic was 35.1% (3). A study has found that asymptomatic patients were younger (37 years vs. 56 years, *P* < 0.001) and had a higher proportion of women (66.7 vs. 31.0%, *P* = 0.002) (1). And the findings of a study highlight that females and children were the predominant groups without symptoms of COVID-19 (4). Due to August 31^st^, 2020, since when asymptomatic infection of COVID-19 draw our attention, 1,740 cases of COVID-19 infection were diagnosed in Guangdong Province according to news released from Guangdong Provincial Central for Disease Control and Prevention (CDC), and the very first COVID-19 case reported in Guangdong Province was back on January 19^th^, 2020, however, the subtype of virus has not been systematically analyzed and no information was reported. Little information was known about asymptomatic patients and their virus genotype, have there been any differences between symptomatic and asymptomatic cases except for clinical symptoms? Therefore, we intend to compare asymptomatic cases with symptomatic cases, so as to find out the prone population of asymptomatic casesand more information about the demographic characteristics of them.

SARS-CoV-2, like other RNA viruses, is prone to genetic evolution while adapting to their new human hosts with the development of mutations over time, resulting in the emergence of multiple variants that may have different characteristics compared to its ancestral strains (5). As Rambaut et al. (6) proposed, we used PANGOLIN classification to categorize virus variants that mentioned in the study for better understanding. For example, SARS-CoV-2 Delta lineages (B.1.617.2) had led to a new wave of outbreak in Guangdong Province lately, which was shorter in incubation period and more virulent (7). According to its transmissibility and virulence, WHO classified SARS-CoV-2 variants into Variants of Concern (VOC) and Variants of Interest (VOI) (8). VOC was defined as a variant which increases in transmissibility, virulence, and decrease in effectiveness of available diagnostics, vaccines, and therapeutics. However, little information is available on whether there is a difference between asymptomatic and symptomatic patients in terms of different SARS-CoV-2 subtypes. The aim of this article is to explore whether there is a significant difference in the occurrence and outcome of asymptomatic persons caused by VOC and VOI infection, and if there are any transmissible and virulent variants not included in VOC that needed to be paid attention to by analyzing the statistical results. We used statistical methods to conduct a retrospective cohort study on 233 confirmed cases whose virus genotypes were known by RNA detection in Guangdong Province of China from September 2020 to February 2021. According to some studies, the ability of asymptomatic infections to spread the virus is not low, and these patients are likely to cause a new round of outbreaks, so finding asymptomatic infections is the key point for early prevention and control of COVID-19 worldwide (9). Meanwhile asymptomatic transmission of SARS-CoV-2 is the Achille's heel of Covid-19 pandemic control through the public health strategies we have currently deployed (10). So we should focus attention and resources on the variants with the highest public health implications to develop more effective immunization strategies.

## Methods

### Study Design

A retrospective cohort study had been carried out at 32 designated hospitals in Guangdong Province. Patients diagnosed with COVID-19 from September 1, 2020 to February 28, 2021 and the genotype of virus confirmed through nucleic acid and gene sequencing technology were included in this study. In order to prevent the spread of COVID-19, the Guangdong COVID-19 Prevention and Control Headquarters had been set up in Guangdong Province. Authorized by the Guangdong Provincial Health Commission, an electronic medical information reporting system (E-System) was built to collect the entire provincial medical data.

### Data Collection

Patients confirmed with SARS-CoV-2 infection during September 1^st^, 2020 to February 28^th^, 2021 been tested for subtype of virus were enrolled in this study, and then went through 14-day follow-up after they had reached criteria of discharge (see below in definitions part). Patients were divided into two groups, the asymptomatic group and the symptomatic group, based on whether any clinical manifestations were shown during whole hospitalization and 14-day follow-up. Data of patient's source country, demography like gender and age, medical history, laboratory tests like blood routine [white blood count (WBC), neutrophils percentage (NE%), lymphocytes percentage (LYMPH%)], inflammation factors [e.g.,: procalcitonin (PCT) and erythrocyte sedimentation rate (ESR)], index of organ function [e.g.,: aspartate transaminase (AST), alanine transaminase (ALT), creatine kinase (CK) and D-dimer], and time point of clinical course like the time patients been tested positive for SARS-CoV-2 RNA, the time they turned negative, the admission time and the discharge time, were extracted from E-system.

For the purpose of ensuring the accuracy and reliability, all the patient's data were reviewed by at least two researchers, and a third researcher adjudicated differences in interpretation if applicable.

### Technology

The method to confirm infection of SARS-CoV-2: a cotton swab was insertedinto patient's pharynx or nasal cavity, then rotated to scrape secreta from mucous membrane and stayed for a few second to obtain a sample (11). Nucleic acid extraction reagent was addedin the sample to destroy the virus and release the nucleic acid, through Reverse transcription Technology (RT), the viral RNA was “reversed” into a specific DNA, that was, cDNA (because the viral RNAstructure was unstable, it was more convenient to convert viral RNA into stable cDNA for detection). And in the process, reverse transcriptase in a nucleic acid test kit played an important role. Reverse transcriptase used dNTP as the substrate, RNA as the template, tRNA (mainly tRNA tryptophan) as the primer, on the 3′- end of tRNA, in the direction of 5′ → 3′, to synthesize a cDNA single strand complementary to RNA template. Then, under the action of reverse transcriptase, the RNA strand was hydrolyzed, and a second DNA strand was synthesized using cDNA as a template. And at this point, the RNA-guided DNA synthesis process was completed. Once reverse transcription was done, cDNA was continuously made in exponential growth with use of PCR. When cDNA was amplified, something called fluorescent-labeled hydrolytic probe in the kit worked at the same time. A fluorescent signal that increased a little bit with each cDNA amplification was emit by the probe, and an increased Ct value of the fluorescent signal was recorded by the PCR detector. Then, the detection results were analyzed according to the Ct values recorded by the detector. Ideally, if there was novel coronavirus in the sample, the Ct values recorded by the detector would form a gradually rising S-shaped curve after the cDNA had been amplified for the number of times booked, and the test result would be positive. If there was no similar S-shaped curve, the test result was negative (12–14).

The method to determine the genotype of infected virus: At first, samples were collected from which virus RNA were extracted, which was consistent with the previous introduction. Next, in order to facilitate detection, it was also needed to transcribe the viral RNA reverse into cDNA. Then, the complete DNA to be tested were mainly broken into 200–500 bp long sequence fragments, and different connectors were added at both ends of these small fragments to construct a single stranded DNA library. When the library was built, the DNA in these libraries would be randomly attached to the channel on the surface of flow cell when passing through the flow cell. There were 8 DNA splices on the surface of each channel, which could support each other in the process of PCR. As for bridge PCR, the connector fixed on the surface of flow cell was used as the template for bridge amplification. After continuous amplification and denaturation cycle, each DNA fragment would finally be concentrated into bundles at their respective positions. Each bundle contained many copies of a single DNA template. The purpose of this process was to amplify the signal strength of the base to meet the signal requirements for sequencing. The sequencing method adopted the method of synthesis and sequencing. DNA polymerase, linker primer and 4 dNTP with base specific fluorescent label were added to the reaction system at the same time (as Sanger sequencing method). The 3′- Oh of these dNTP was protected by chemical methods, so only one dNTP could be added at a time. After dNTP was added to the synthetic chain, all unused free dNTP and DNA polymerase would be washed off. Then, the buffer required to excite fluorescence was added, the fluorescence signal was excited by laser, and the fluorescence signal was recorded by optical equipment. Finally, the optical signal was transformed into sequencing base by computer analysis. Finally, the measured DNA sequence was compared with the existing database and a test report was issued to know the genotype of the infected virus (15, 16).

### Definitions

Asymptomatic patient refers to individuals with laboratory-confirmed SARS-CoV-2 infection but without symptoms throughout their entire course of infection, or after 14 days of follow up (1).

The criteria of discharge: (1) body temperature reached normal for more than 3 days; (2) respiratory symptoms improved significantly; (3) improvement in lung computed tomography scans (CT); (4) negative SARS-CoV-2 RNA tests for 2 consecutive times (24-h interval) (11).

The criteria for nucleic acid turning negative were as follows: the patient's symptoms had basically disappeared, CT examination showed that the lung lesions had been basically absorbed, and more than two consecutive (intervals of more than 24 h) nucleic acid tests were negative, And the interval between positive nucleic acid test and consecutive (intervals of more than 24 h) nucleic acid tests was time of SARS-CoV-2 RNA turning negative (17). Time of hospitalization refers to interval between admission and discharge (18).

WHO has been constantly monitoring and assessing the variantsofSRAS-CoV-2, and variants were categorized as Variants of Interest (VOI) and Variants of Concern (VOC) in accordance to its transmissibility and toxicity (8).

VOI are identified as SARS-CoV-2 variants with genetic changes that are predicted or known to affect virus characteristics such as transmissibility, disease severity, immune escape, diagnostic or therapeutic escape and identified to cause significant community transmission or multiple COVID-19 clusters, in multiple countries with increasing relative prevalence alongside increasing number of cases over time, or other apparent epidemiological impacts to suggest an emerging risk to global public health. VOI mainly include lineage B.1.427, lineage B.1.429, lineage P.2, lineage B.1.525, lineage P.3, lineage B.1.526, lineage B.1.617.1, and lineage C.37 so far.

VOC are identified as SARS-CoV-2 variants that meet the definition of a VOI and have been demonstrated to be associated with one or more of the following changes at a degree of global public health significance: Increase in transmissibility or detrimental change in COVID-19 epidemiology; OR increase in virulence or change in clinical disease presentation; OR decrease in effectiveness of public health and social measures or available diagnostics, vaccines, therapeutics. VOC mainly include lineage B.1.1.7, lineage B.1.351, lineage B.1.351.2, lineage B.1.351.3, lineage P.1, lineage P.1.1, lineage P.1.2, lineage B.1.617, lineage AY.1, lineage AY.2, and lineage AY.3 so far.

### Statistical Analysis

All data were analyzed by SPSS26.0 software. Exploratory analysis was used for normality test and *P* < 0.05 suggests that the data did not obey the normal distribution. The categorical variables were expressed in frequency (percentage) and Chi-square test or Fisher's exact tests were used for comparison between the two groups (the asymptomatic and the symptomatic) and two-sided *p* of < 0.1 was considered statistically significant. The normal distribution of continuous variables was expressed as mean ± standard deviation, and the skew distribution was expressed as median with interquartile range (IQR). Independent sample *T*-test or non-parametric test was used, and two-sided *p* of < 0.1 was considered statistically significant.

## Results

### Demographic and Basic Clinical Characteristics of the Study Population

As the Table 1 shown, a total of 233 patients diagnosed with COVID-19 were enrolled in this study, composed of 83 asymptomatic patients and 150 symptomatic patients. Eighty three asymptomatic patients had younger average age (32.71 ± 12.17 vs. 36.73 ± 12.55, *P* = 0.019), less comorbidities (8.4 vs. 20.0%, *P* = 0.021) such as hypertension (2.4 vs. 9.3%, *P* = 0.021) and chronic liver disease (1.2 vs. 8.7%, *P* = 0.045), but higher in chronic lung disease (4.8 vs. 0.7%, *P* = 0.056) when compared to symptomatic group. There was no significant difference in sex, comorbidity of cardiovascular, cerebrovascular, chronic kidney diseases and the time of SARS-CoV-2 RNA turning negative between the two groups.

**Table 1:** Clinical characteristics and time of SARS-CoV-2 RNA turning negative in asymptomatic and symptomatic groups.

	**Asymptomatic**	**Symptomatic**	***p*-value**
Gender (male)	75.9% (63)	75.3% (113)	0.923
Age	32.71 ± 12.17	36.73 ± 12.55	0.019
**Comorbidity**			
Any	8.4% (7)	20% (30)	0.021
Diabetes	0	1.3% (2)	
Hypertension	2.4% (2)	9.3% (14)	0.083
Cardiovascular disease	1.2% (1)	4.0% (6)	0.426
Cerebrovascular disease	0	0	
Chronic kidney disease	0	2.0% (3)	
Chronic lung disease	4.8% (4)	0.7% (1)	0.056
Chronic liver disease	1.2% (1)	8.7% (13)	0.045
History of cancer	0	0	
**Variants**			0.612
A	4.8% (4)	4.0% (6)	
B	95.2% (79)	95.3% (143)	
C	0	0.7% (1)	
**Laboratory test**			
Oxygenation index	451.00	450.00	0.320
SpO2%	99.00	99.00	0.661
WBC	6.155	5.280	0.002
NE%	55.65%	60.40%	0.019
LYMPH%	31.30%	27.95%	0.011
PCT	0.055	0.108	0.021
ESR	11.00	10.00	0.820
AST	21.11	21.80	0.114
ALT	21.00	23.53	0.029
CK	53.50	71.00	0.025
DDI	0.22	85.00	<0.001
Time of SARS-CoV-2 RNA turn negative	18.00	17.00	0.937

*Symptomatic group significantly lower in WBC count and lymphocyte count percentage but higher in pct, ALT, CK and DDI than asymptomatic group*.

### Clinical Laboratory Tests of the Study Population

Patients tested positive for SARS-CoV-2 RNA shall be admitted to designated hospital as soon as possible, and the day they got admission, laboratory tests as blood routine, inflammation factors, index of organ function level should be tested immediately for indication of disease severity and guidance of therapy. When compared to the symptomatic group, the asymptomatic group was lower in NE% (55.65 vs. 60.4%, *P* = 0.019), PCT (0.055 vs. 0.108, *P* = 0.021), ALT (21 vs. 23.53, *P* = 0.029), CK (53.5 vs. 71, *P* = 0.025) and DDI (0.22 vs. 85, *P* < 0.001), but has higher WBC count (6.155 vs. 5.280, *P* = 0.002) and LYMPH% (31.3% vs. 27.95, *P* = 0.011) (Table 1).

### Virus Genotypes Analysis

Of the 223 COVID-19 patients in the study, 10 were infected with virus genotypes from PANGOLIN cluster A, 222 were infected with virus genotypes from PANGOLIN B cluster and 1 was infected with virus genotype of PANGOLIN C cluster. Therefore, virus genotypes from PANGOLIN B cluster accounted for more than 95% of the total and were the focus of this study (Table 1).

**Figure 1:**
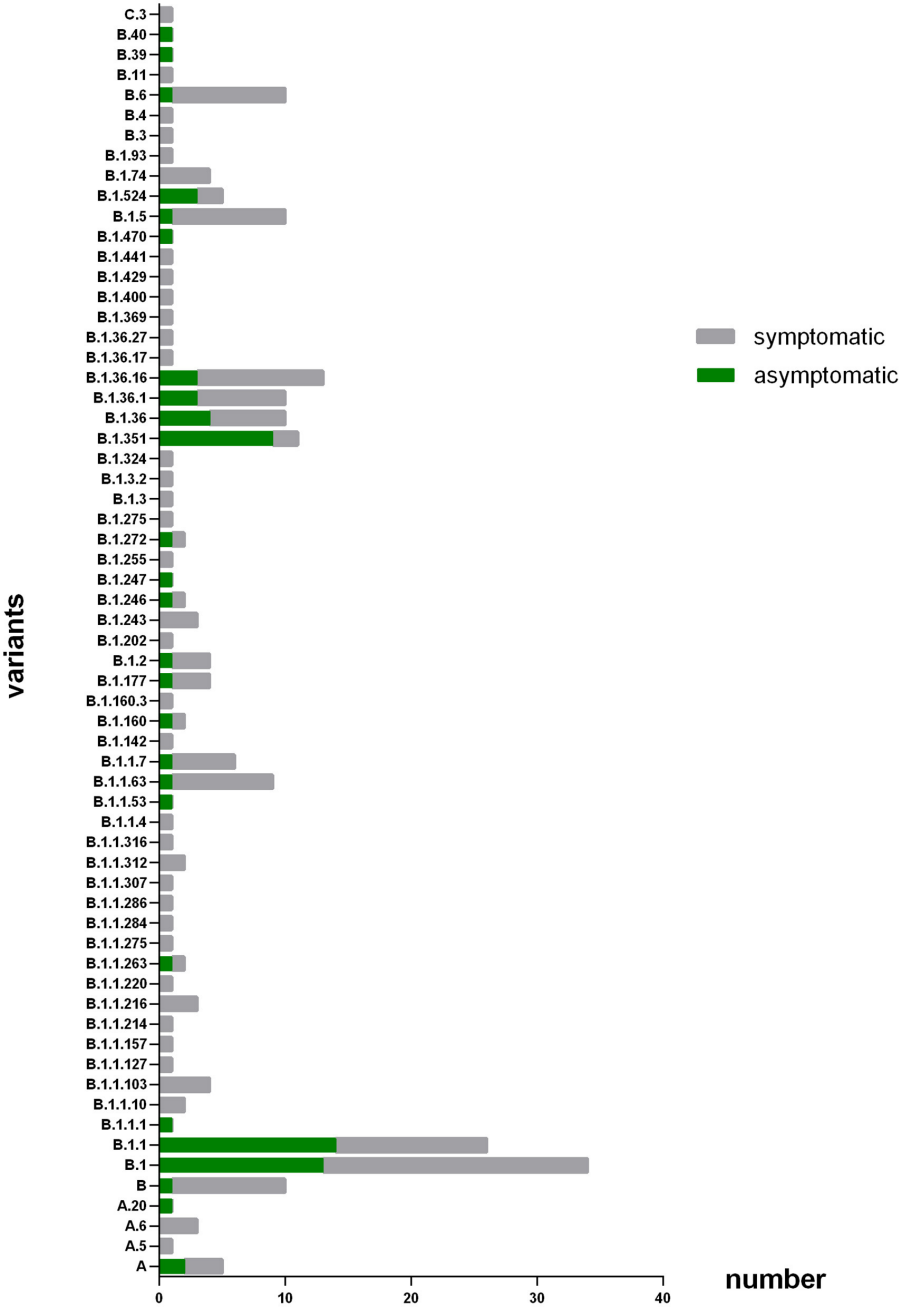
Number of patients with different variants. The X axis refers to number of patients that infected with different subtype of virus; the Y axis refers to the different virus subtype that involved in this study. The coronavirus variants that patients enrolled in this study get infected major concentrated in B, B,1, B,1,1, B,1,1,63, B,1,1,7, B.1.351, B.1,36, B.1.36.1, B.1.36.16, B.15, B.1.524, and B.6.

As Figure 1 shown, the patients were infected with 63 different virus genotypes, of which 4 were from PANGOLIN A cluster, 58 were from PANGOLIN B cluster, and 1 was from PANGOLIN C cluster. And among the 58 virus genotypes from PANGOLIN B cluster, there were 12 kinds of major variants from PANGOLIN B cluster (*n* ≥ 5): B, B.1, B.1.1, B.1.1.63, B.1.1.7, B.1.351, B.1.36, B.1.36.1, B.1.36.16, B.1.5, B.1.524, and B.6. Among these major genotypes, patients infected with lineage B.1.1 (53.8%),B.1.351 (81.8%), B.1.524 (60%) were mainly asymptomatic, while patients infected with lineage B (90.0%), B.1 (61.8%), B.1.1.63 (88.9%), B.1.1.7 (83.3%), B.1.36 (60.0%), B.1.36.1 (70.0%), B.1.36.16 (76.9%), B.1.5 (90.0%), and B.6 (90.0%) were mainly symptomatic.

Lineage B.1.1.7 (also known as the Alpha variants) and lineage B.1.351 (also known as the Beta variants) are cataloged as variants of concern (VOC) according to definition proposed by WHO, patients infected with B.1.1.7 were mainly symptomatic, had higher proportion of comorbidities (66.7%) especially chronic liver disease (50%), and had higher ALT level (52.750). For those patients who infected with lineage B.1.351, they were mainly asymptomatic and higher in lymphocyte percentage (60.7%) while lower in CK (46.778) and DDI (0.402) level.

### Source Country of the Asymptomatic and Symptomatic Patients

**Figure 2:**
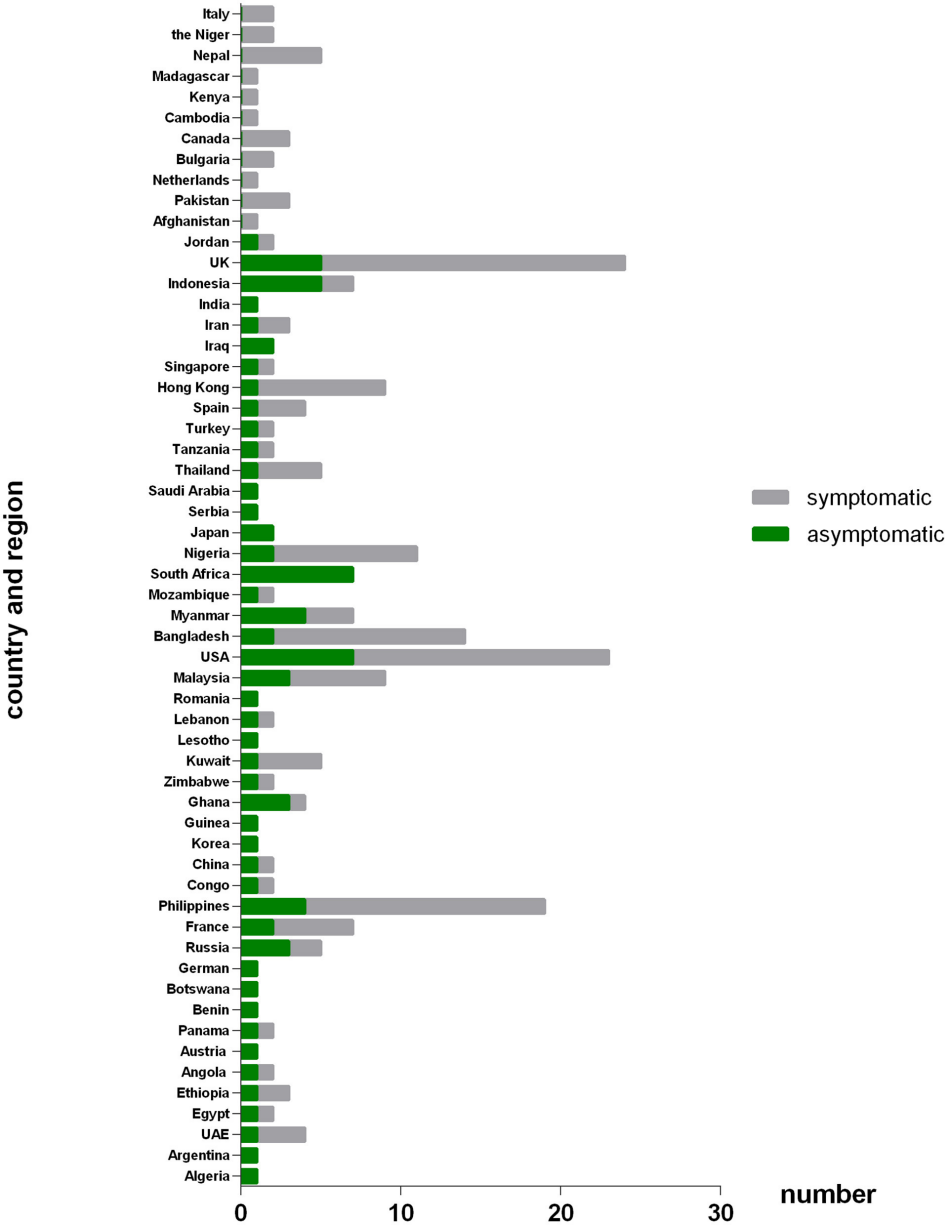
Number of patients originated from different countries or regions between asymptomatic and symptomatic groups. The X axis refers to number of patients that come from different countries or regions; the Y axis refers to the different countries or regions that involved in this study. Patients originate from Russia, Ghana, Myanmar, Indonesia and especially South Africa was dominated by asymptomatic cases. While patients originate from France, Philippines, Kuwait, Malaysia, America, Bangladesh, Nigeria, and HK-China were dominated by symptomatic cases.

As Figure 2 shown, 233 COVID-19 patients in this study were from 57 different countries or regions. And the patients were mainly associated with Russia, France, Philippines, Ghana, Kuwait, Malaysia, United State of America (USA), Bangladesh, Myanmar, South Africa, Nigeria, Hong Kong (Chinese special administrative region), Indonesia and United Kingdom (UK). Of the patients from Russia, Ghana, Myanmar, Indonesia and especially South Africa, the asymptomatic group were the majority. While of the patients from France, Philippines, Kuwait, Malaysia, USA, Bangladesh, Nigeria, and Hong Kong, the symptomatic were the majority.

### Laboratory Examinations in Total and Variants Infected Patients

As Table 2 shown, when compared to the other groups, patients with lineage B had higher CK level (157.667 vs. 105.885, *P* = 0.003) and lower DDI level (604.376 vs. 6280.456, *P* = 0.081). Patients with lineage B.1were older than the other groups (39.5 vs. 35.3, *P* = 0.054) and lower ALT (21.891 vs. 26.647, *P* = 0.097). Lineage B.1.1 had higher rate of asymptomatic patients (53.8 vs. 35.6%, *P* = 0.040) and lower ESR level (8.4 vs. 13.43, *P* = 0.091). Patients with lineage B.1.1.63 had higher AST (53.991 vs. 24.703, *P* = 0.001) and ALT (54.171 vs. 26.647, *P* = 0.017). Patients with lineage B.1.1.7 had higher comorbidities rate (66.7 vs. 15.9%, *P* = 0.004), especially in chronic liver disease (50.0 vs. 6.0%, *P* < 0.001), and higher SpO2% (99.50 vs. 98.49%, *P* = 0.007) and ALT (52.75 vs. 26.647, *P* = 0.035). Lineage B.1.351 had higher rate of asymptomatic patients (81.8 vs. 35.6%, *P* = 0.003), higher LYMPH% (60.7 vs. 30.3%, *P* < 0.001), lower NE% (47 vs. 58.5%, *P* = 0.008), CK (46.778 vs. 105.885, *P* = 0.022), and DDI (0.402 vs. 6280.456, *P* = 0.046). Lineage B.1.36 hadlower oxygenation index (191.900 vs. 371.970, *P* = 0.004). Lineage B.1.36.16 had higher ALT level (36.217 vs. 26.647, *P* = 0.089). Lineage B.1.5 had lower SpO2% (97.5 vs. 98.49%, *P* = 0.041), and DDI (812.417 vs. 6280.456, *P* = 0.004). Patients with lineage B.1.524 had higher oxygenation index (472.800 vs. 371.970, *P* = 0.095), ESR (43.5 vs. 13.43, *P* = 0.030) and lower CK level (23.574 vs. 105.885, *P* = 0.088). And those with lineage B.6 were older than the other groups (43.10 vs. 35.30, *P* = 0.037), had higher hypertension rate (30.0 vs. 6.9%, *P* = 0.020), higher PCT level (42.109 vs. 14.317, *P* = 0.013), ALT level (35.040 vs. 26.647, *P* = 0.066), and lower DDI level (967.051 vs. 6280.456, *P* = 0.004).

**Table 2:** Clinical characteristics and time of SARS-CoV-2 RNA turning negative in total and variants infected patients (*n* ≥ 5).

	**Total**	**B**	**B.1**	**B.1.1**	**B.1.1.63**	**B.1.1.7**	**B.1.351**	**B.1.36**	**B.1.36.1**	**B.1.36.16**	**B.1.5**	**B.1.524**	**B.6**
Number	233	10	34	26	9	6	11	10	10	13	10	5	10
Asymptomatic	83 (35.60%)	1 (10.0%)	13 (38.2%)	14 (53.8%)^*^	1 (11.1%)	1 (16.7%)	9 (81.8%)^*^	4 (40.0%)	3 (30.0%)	3 (23.1%)	1 (10.0%)	3 (60.0%)	1 (10.0%)
Gender (male)	0.755	0.90	0.559	0.846	0.889	1	1	0.80	0.50	0.923	0.70	0.60	1
Age	35.30	37.80	39.50^*^	34.15	30.78	35.50	37.18	33.00	42.70^*^	38.23	33.50	40.00	43.10^*^
**Comorbidity**													
Any	0.159	0.30	0.118	0.038	0	0.667^*^	0.091	0.30	0	0.077	0.10	0	0.30^*^
Diabetes	0.009	0	0	0	0	0	0	0	0	0	0	0	0
Hypertension	0.069	0.20	0.088	0	0	0.167	0.091	0.10	0	0.077	0	0	0.30^*^
Cardiovascular disease	0.030	0	0	0.038	0	0.167	0.091	0	0	0	0	0	0.10
Cerebrovascular disease	0	0	0	0	0	0	0	0	0	0	0	0	0
Chronic kidney disease	0.013	0	0.029	0.038	0	0	0	0	0	0	0.10	0	0
Chronic lung disease	0.021	0.10	0	0	0	0.167	0	0.10	0	0	0	0	0
Chronic liver disease	0.060	0	0.029	0.038	0	0.50^*^	0	0.10	0	0	0	0	0
History of Cancer	0	0	0	0	0	0	0	0	0	0	0	0	0
**Laboratory test**													
Oxygenation index	371.97	387.15	397.30	393.27	420.97	449.50	456.00	191.90^*^	413.98	406.08	421.60	472.80^*^	369.500
SpO2%	98.49	97.93	98.40	98.56	98.44	99.50^*^	98.64	98.80^*^	98.20	98.62	97.89^*^	98.14	98.62
WBC	5.995	5.924	6.530	5.309	6.916	6.973	6.453	5.070	5.261	6.654	5.674	6.560	5.725
NE%	0.585	0.574	0.618	0.596	0.594	0.658	0.470^*^	0.608	0.562	0.653	0.610	0.618	0.541
LYMPH%	0.303	0.290	0.267	0.290	0.289	0.218	0.607^*^	0.309	0.254	0.235	0.293	0.270	0.342
PCT	14.32	32.74	11.53	19.44	0.041	-	0.281	0.100	0.097	0.077	28.758	0.045	42.109^*^
ESR	13.430	6.60	9.556	8.400^*^	15.500	-	18.889	12.000	12.400	19.667	32.667	43.500^*^	11.500
AST	24.703	23.07	22.487	22.016	53.991^*^	46.500	21.578	21.000	23.640	30.100	25.083	18.175	23.600
ALT	26.647	29.95	21.891^*^	24.376	54.171^*^	52.750^*^	25.356	21.000	24.700	36.217^*^	26.300	23.075	35.040^*^
CK	105.885	157.667^*^	69.195	274.206	120.500	82.000	46.778^*^	65.333	47.600	54.328	111.614	23.574^*^	77.840
DDI	6280.456	604.376^*^	38268.660	2396.986	66.000	-	0.402^*^	0.333	102.506	896.328	812.417^*^	0.257	967.051^*^
Time of	17	19	17	18.00	12.5	24	26	21	18	13.5	11.50	41	14
SARS-CoV-2 RNA turning negative	(11.00–25.00)	(13.50–27.00)	(10.00–24.00)	(10.50–22.50)	(5.00–19.00)	(15.00–31.50)	(18.50–27.25)^*^	(11.00–27.75)	(13.50–29.25)	(4.25–27.75)	(8.75–18.25)	(22.00–46.00)^*^	(11.00–19.00)

“*” *Means significant difference (P < 0.1). When compared to the whole other groups, patients with lineage B had higher CK level (157.667 vs. 105.885, P = 0.003) and lower DDI level (604.376 vs. 6280.456, P = 0.081), Patients with lineage B.1 were older than the other groups (39.5 vs. 35.3, P = 0.054) and lower ALT (21.891 vs. 26.647, P = 0.097). Lineage B.1.1 had higher rate of asymptomatic patients (53.8 vs. 35.6%, P = 0.040) and lower ESR level (8.4 vs. 13.43,P = 0.091). Patients with lineage B.1.1.63 had higher AST (53.991 vs. 24.703, P = 0.001) and ALT (54.171 vs. 26.647, P = 0.017). Patients with lineage B.1.1.7 had higher comorbidities rate (66.7 vs. 15.9%, P = 0.004), especially in chronic liver disease (50.0 vs. 6.0%, P < 0.001), and higher SpO2% (99.50 vs. 98.49%, P = 0.007) and ALT (52.75 vs. 26.647, P = 0.035). Lineage B.1.351 had higher rate of asymptomatic patients (81.8 vs. 35.6%, P = 0.003), higher LYMPH% (60.7 vs. 30.3%, P < 0.001), lower NE% (47 vs. 58.5%, P = 0.008), CK (46.778 vs. 105.885, P = 0.022), and DDI (0.402 vs. 6280.456, P = 0.046). Lineage B.1.36 had lower oxygenation index (191.900 vs. 371.970, P = 0.004). Lineage B.1.36.16 had higher ALT level (36.217 vs. 26.647, P = 0.089). Lineage B.1.5 had lower SpO2% (97.5 vs. 98.49%, P = 0.041), and DDI (812.417 vs. 6280.456, P = 0.004). Patients with lineage B.1.524 had higher oxygenation index (472.800 vs. 371.970, P = 0.095), ESR (43.5 vs. 13.43, P = 0.030) and lower CK level (23.574 vs. 105.885, P = 0.088). And those with lineage B.6 were older than the other groups (43.10 vs. 35.30, P = 0.037), had higher hypertension rate (30.0 vs. 6.9%,P = 0.020), higher PCT level (42.109 vs. 14.317, P = 0.013), ALT level (35.040 vs. 26.647, P = 0.066), and lower DDI level (967.051 vs. 6280.456, P = 0.004)*.

### Time of SARS-CoV-2 RNA Turning Negative or Hospitalization in Total and Variants Infected Patients

**Figure 3:**
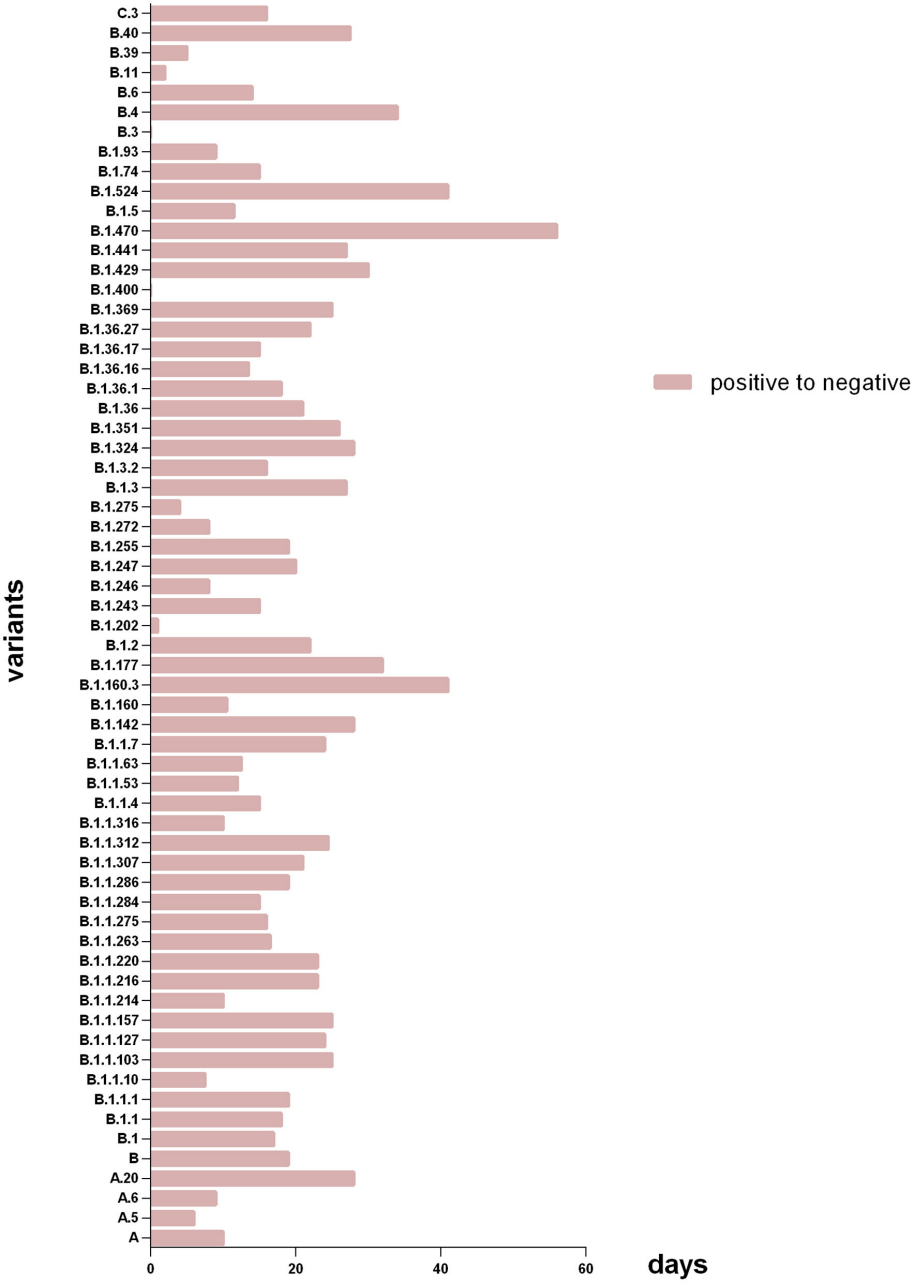
Days of patients with different variants from SARS-CoV-2 RNA positive to negative. The X axis refers to days of patients with different subtype spent in SARS-CoV-2 RNA turning negative; the Y axis refers to the different virus subtype that involved in this study. Patients with lineage B.1.470 had the longest median time of SARS-CoV-2 RNA turning negative, and the shortest were B.3 and B.1.400.

**Figure 4:**
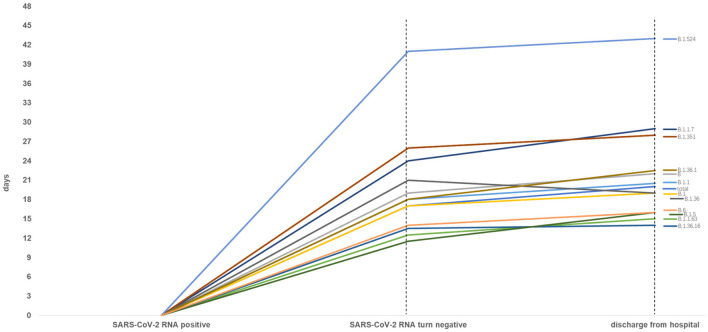
Time from SARS-CoV-2 RNA positive to negative among total and other variants infected patients (*n* ≥ 5). Patients with lineage B.1.524 significantly spent longer time in SARS-CoV-2 RNA turning negative and hospitalization.

As Tables 2, 3 shown, the total median time of SARS-CoV-2 RNA turning negative and hospitalization were 17 days (IQR 11–25) and 20 days (IQR 14.5–28) relatively. When compared to the whole other groups, we observed that patients with lineage B.1.351 had a longer time of SARS-CoV-2 RNA turning negative (26 vs. 17, *P* = 0.085) and hospitalization (28 vs. 20, *P* = 0.085). Patients infected with lineage B.1.36.16 (14 vs. 20, *P* = 0.063) and B.6 (16 vs. 20, *P* = 0.073) had a shorter time of hospitalization respectively. Patients infected with Lineage B.1.524 had a much longer time in SARS-CoV-2 RNA turning negative (41 vs. 17, *P* = 0.007) and hospitalization (43 vs. 20, *P* = 0.004), which could also see in Figures 3, 4.

**Table 3:** Comparison of asymptomatic and time of SARS-CoV-2 RNA turning negative between total and variants infected patients (*n* ≥ 5).

	**Age**	***p*-value**	**Asymptomatic**	***p*-value**	**Time of SARS-CoV-2**	***p*-value**	**Time of**	***p*-value**
					**RNA turningnegative**		**hospitalization**	
Total (*n* = 233)	35.3		83 (35.60%)		17 (11.00–25.00)		20 (14.50–28.00)	
VOC (α+β) (*n* = 17)	36.59		10 (58.8%)		26 (21.00–28.00)		28 (23.50–30.50)	
Non-VOC (*n* = 137)	37.27	0.854	43 (31.4%)	0.025	17 (10.00–24.00)	0.027	19 (14.00–26.00)	0.010
B (*n* = 10)	37.8	0.523	1 (10.0%)	0.164	19 (13.50–27.00)	0.577	22 (16.50–31.75)	0.545
B.1 (*n* = 34)	39.5	0.054	13 (38.2%)	0.731	17 (10.00–24.00)	0.627	19 (14.00–26.25)	0.436
B.1.1 (*n* = 26)	34.15	0.558	14 (53.8%)	0.040	18.00 (10.50–22.50)	0.906	20.50 (15.00–24.50)	0.860
B.1.1.63 (*n* = 9)	30.78	0.318	1 (11.1%)	0.226	12.5 (5.00–19.00)	0.140	15 (8.00–24.50)	0.152
B.1.1.7 (*n* = 6)	35.5	0.973	1 (16.7%)	0.582	24 (15.00–31.50)	0.206	29 (21.75–33.50)	0.119
B.1.351 (*n* = 11)	37.18	0.595	9 (81.8%)	0.003	26 (18.50–27.25)	0.085	28 (23.00–30.00)	0.085
B.1.36 (*n* = 10)	33.00	0.536	4 (40.0%)	0.999	21 (11.00–27.75)	0.694	19 (13.25–30.50)	0.823
B.1.36.1 (*n* = 10)	42.70	0.090	3 (30.0%)	0.966	18 (13.50–29.25)	0.646	22.5(14.75–26.75)	0.897
B.1.36.16 (*n* = 13)	38.23	0.237	3 (23.1%)	0.50	13.5 (4.25–27.75)	0.273	14 (8.00–24.50)	0.063
B.1.5 (*n* = 10)	33.50	0.757	1 (10.0%)	0.164	11.50 (8.75–18.25)	0.142	16 (12.75–21.25)	0.110
B.1.524 (*n* = 5)	40.00	0.349	3 (60.0%)	0.351	41 (22.00–46.00)	0.007	43 (31.00–52.00)	0.004
B.6 (*n* = 10)	43.10	0.037	1 (10.0%)	0.164	14 (11.00–19.00)	0.214	16 (12.50–20.75)	0.073

*Patients infected with variant B.1.1 and B.1.351 had more asymptomatic cases in this study. Patients infected with variant B.1.351 and B.1.524 spent longer time in SARS-CoV-2 RNA turn negative and hospitalization. Patients infected with lineage B.1.36.16 (14 vs. 20, P = 0.063) and B.6 (16 vs. 20, P = 0.073) had a shorter time of hospitalization, respectively*.

As Figure 3 shown, among all genotypes in this study, the patients with lineage B.1.470 had the longest median time of SARS-CoV-2 RNA turning negative, and the shortest were B.3 and B.1.400. And among the major genotypes from PANGOLIN B cluster (*n* ≥ 5) in the study, patients with lineage B.1.36.16 had the minimum median time of SARS-CoV-2 RNA turning negative.

As Figure 4 shown, when compared to the total, the patients with lineage B, B.1.1, B.1.1.7, B.1.351, B.1.36.1, B.1.524 had longer median time of SARS-CoV-2 RNA turning negative and hospitalization, and patients with lineage B.1.1.63, B.1.36.16, B.1.5 and B.6 had a shorter median time of SARS-CoV-2 RNA turning negative and hospitalization. Whereas, the median time of SARS-CoV-2 RNA turning negative of patients with lineage B.1.36 was equal to the total, the median time of hospitalization was shorter to the total.

## Discussion

Asymptomatic cases of COVID-19 are more often been reported over countries and regions, the proportion of asymptomatic cases out of positive SARS-CoV-2 RNA infectors could range from 4 to 91.7% quoted from related studies (1, 9, 19). These studies reported that asymptomatic cases were younger and had a higher proportion of women (see previous), lower in monocytes and alanine aminotransferase compared to symptomatic cases. The prevalence of asymptomatic case reported from those studies varies a lot, also lack of clinical outcome and message of virus subtype. To verify the prevalence and characteristics of asymptomatic cases, this study was conducted and to further explore the impact of different virus subtype could do to clinical manifestation of COVID-19 patients.

The proportion of asymptomatic cases in this study was 35.6%. Previous reports had suggested that asymptomatic cases can also be communicable (20–23) and more easily to cause popular transmission in community than symptomatic cases since the public are much alert to people who have symptoms like cough, fever or etc. that rather attract attention and have been detected earlier. But patients with no symptom manifested could relax the guard instead. Thus, recognizing asymptomatic patient at an early stage and taking action to it plays an important role in prevention and control of COVID-19 nowadays.

Our data revealed that asymptomatic patients comprised majorly of Mongolian, and were younger, had lower rate in comorbidities such as hypertension and chronic liver disease. As for laboratory test, we discovered that asymptomatic patients were higher in WBC count and lymphocyte, but lower in pct, ALT and DDI compared to symptomatic patients. The results indicated that younger patients with less comorbidities prone to be asymptomatic when infected with novel coronavirus, due to their stronger immune function, they had milder inflammation response and less damage to organ function.

Qin, Wang, Qu et al. (24–26) suggested that decreased lymphocyte account indicated a severe phenotype of inflammation or disease which leads to longer hospitalization time. And there are studies noticed that higher D-Dimer level has a positive relationship to severity of COVID-19 disease and hospitalization (26–28). Laboratory tests in our study recommend that inflammatory response was milder in asymptomatic patients and less in liver damage that consist with lower comorbidity especially chronic liver disease mentioned before, which could explain the slight clinical symptoms.

Though some studies reported higher proportion of female in asymptomatic cases (1, 4), no significant differences in gender between symptomatic and asymptomatic group were observed in this study, further study of larger sample size was needed for verification.

Nevertheless, no significant difference shown in time of positive SARS-CoV-2 RNA turn negative between asymptomatic and symptomatic patients, this reminds a similar viral shedding between the two groups, and there is report suggested viral load between symptomatic and asymptomatic patients had no difference neither (29). But what makes this significant difference in clinical features between symptomatic and asymptomatic patients left in a mystery, and we wonder whether different viral subtype could be relevant.

SARS-CoV-2 RNA is easily getting mutate during its duplication, and patients infected with different variants of coronavirus could be various in pattern of clinical manifestation, course of disease and prognosis, which brings huge difficulty to diagnosis, therapy and prevention in clinics.

We managed to gain the 233 patient's nasopharyngeal or oral swab, those specimens went through rt-PCR test to clear its variants that got infected, and clarified with PANGOLIN lineage, among which, only 10 patients were infected with PANGOLIN A cluster, 1 patient was infected with PANGOLIN C cluster, and the other 222 patients were infected with PANGOLIN B cluster. Most of the patients enrolled in this study were infected with variants from PANGOLIN B cluster, and based on the difference of spike mutation of SRAS-CoV-2, PANGOLIN B cluster was further divided into different variants.

From our data, the variants patients got infected majorly concentrated on lineage B, B.1, B.1.1, B.1.1.63, B.1.1.7, B.1.351, B.1.36, B.1.36.1, B.1.36.16, B.1.5, B.1.524 and B.6. Among them, patients infected with lineage B.1.1, B.1.351 and lineage B.1.524 were dominate by asymptomatic infectors. Due to the few amounts of patients infected with other lineages, the 12 lineages above were chosen separately as subgroup for further analysis, comparison between every single lineage subgroup and other subgroups were performed.

Among 12 variants above, B.1.1.7 and B.1.351 were cataloged as VOC, and the other 10 variants neither VOC nor VOI. Time of SARS-CoV-2 RNA turning negative in VOC group was longer than in total. 83.3% of patients infected with B.1.1.7 (the Alpha variant) were symptomatic, had higher comorbidity rate and ALT level, which comply with common know. However, patients infected with B.1.351 were mainly asymptomatic (81.8%), and had higher level of lymphocyte account, ALT and DDI, spent longer time in SARS-CoV-2 RNA turning negative. The elevated ALT and DDI level could be explained by its stronger toxicity, but the high asymptomatic ratio, which disobeyed its characteristics, bring us confusion. With the increasing number of COVID-19 patients, the public's alert to this pandemic is growing, and detection of COVID-19 is more intensive and rigorous. For example, people who take public transportations shall go through examinations by local staff from Centers for Disease Control (CDC), and those had clinical symptoms such as fever, cough or etc. are much more difficult to pass these examinations, infected people with milder symptoms or even lack of them are more possible to escape and travel to other country or region, and this may explain why patients infected with B.1.351 enrolled in this study are majorly asymptomatic.

When compared to the total, we observed that 60% patients infected with lineage B.1.524 were asymptomatic, they hada longer time in SARS-CoV-2 RNA turning negative and hospitalization, even longer than VOC group (B.1.1.7 + B.1.351), prompted B.1.524 has longer viral shedding period. There is a study reported that SARS-CoV-2 from third wave clusters in Malaysia was dominant by local lineage B.1.524 (30). The long viral shedding time in lineage B.1.524 madeit longer to turn SARS-CoV-2 RNA negative, indicated a wilder time range to cause transmission especially from asymptomatic patients. Asymptomatic infection would decrease our alertness and more easily to spread in public, difficulties in epidemic prevention would increase and more medical resource would be consumed. For symptomatic patients infected with B.1.524, the long time in SARS-CoV-2 RNA turning negative and hospitalization indicate greater viral load that could be much burden to patient's organ function and aggravate poor prognosis.

Lineage B.1.524 shall attract more concentration, nonetheless that this study was lack of enough sample and data to analyze the transmissibility and toxicity of lineage B.1.524, thus further multi-center study was expected to be conducted.

There are limitations in our study. We collected data of SARS-CoV-2 RNA turn negative time and speculate its similar viral shedding time among different variants, but no specimens had been tested for viral load at patients got admission, and samples of variants are in deficiency. We expect to collect data of viral load, virus subtype and information of spread, analysis could be made to compare the toxicity of different variants.

In conclusion, asymptomatic cases are prone to develop in patients with younger age, comparatively good immune and organ function and less comorbidities, especially those who infected with lineage B.1.1 and B.1.524. More attention should be paid to lineage B.1.524 for it can cause poor prognosis and significantly prolong the SARS-CoV-2 RNA negative conversion time and hospitalization.

## Data Availability Statement

The original contributions presented in the study are included in the article/supplementary materials, further inquiries can be directed to the corresponding author/s.

## Ethics Statement

The studies involving human participants were reviewed and approved by the Institutional Review Board of Guangdong Health Commission and Guangdong Provincial People's Hospital (No. GDREC2020028H). Written informed consent from the participants' legal guardian/next of kin was not required to participate in this study in accordance with the national legislation and the institutional requirements.

## Author Contributions

MF had the idea for and designed the study. HX, C-yX, P-hL, and J-fS had full access to all data in the study and took responsibility for the integrity of the data. HX, C-yX, P-hL, and Z-lJ were responsible for data collection and data analysis. MF, HX, C-yX, and P-hL contributed to writing of the report. BH and XL polished the article. All authors contributed to data acquisition and data interpretation, reviewed, and approved the final version.

## Funding

This study was supported by the Health Commission of Guangdong Province, the Department of Science and Technology of Guangdong Province, and the Special Project on Emergency Response to Control of Novel Coronavirus Infection of Guangdong Province (No. 2020B1111330006).

## Conflict of Interest

The authors declare that the research was conducted in the absence of any commercial or financial relationships that could be construed as a potential conflict of interest.

## Publisher's Note

All claims expressed in this article are solely those of the authors and do not necessarily represent those of their affiliated organizations, or those of the publisher, the editors and the reviewers. Any product that may be evaluated in this article, or claim that may be made by its manufacturer, is not guaranteed or endorsed by the publisher.
